# Age-Related Differences between Maximum Flight Height of Basic Skills on Floor, Beam and Vault and Physical Condition of Youth Female Artistic Gymnasts

**DOI:** 10.3390/sports11050100

**Published:** 2023-05-08

**Authors:** Christoph Schärer, Luca Reinhart, Klaus Hübner

**Affiliations:** Swiss Federal Institute of Sport Magglingen, Hauptstrasse 247, 2532 Magglingen, Switzerland

**Keywords:** women’s gymnastics, adolescent, children, lower body power, reactive strength, apparatus

## Abstract

In women’s artistic gymnastics, difficult elements with great flight heights have to be performed on the apparatuses. However, the importance of the physical condition for generating flight height and its development with age remains elusive. Therefore, we investigated the age-related differences of lower body power, reactive strength, 20 m sprint speed, flight heights (basic elements on beam and floor) and run-up speed on the vault of 33 youth female gymnasts. Further, we calculated correlations between all parameters separately for different age groups (7–9 y; 10–12 y; 13–15 y). We found larger differences between the age groups 7–9 y and 10–12 y than between 10–12 y and 13–15 y on the apparatuses (10–12 y vs. 7–9 y: +23% to +52%; 13–15 y vs. 10–12 y: +2% to +24%) and for physical conditioning variables (10–12 y vs. 7–9 y: +12 to +24%; 13–15 y vs. 10–12 y: + 5% to +16%). The correlations between flight heights and physical condition were the lowest for age group 7–9 y (r: from −0.47 to 0.78; 10–12 y: r: from −0.19 to 0.80; 13–15 y: r: from −0.20 to 0.90). An optimal application of the physical condition to enhance the gymnastics-specific performance (e.g., flight height) is strongly age-dependent. Regular monitoring of jumping abilities and the derivation of training recommendations can accelerate this development and the future performance of young athletes.

## 1. Introduction

In international women’s artistic gymnastics, the level is constantly rising. In order to develop the technical skills and the prerequisite physical conditions, high training volumes are already required in childhood and adolescence [1].

High levels of muscular power, maximum strength, speed and flexibility are required to perform the difficult elements on the four apparatuses [2]. It is noteworthy that on three of the four apparatuses, the ability to jump effectively is crucial. Therefore, the level of lower body power is fundamental to be able to learn new skills [3] and for a successful performance at competitions.

During competitive routines, difficult acrobatic elements must be performed as close as possible to perfection. To ensure that these can be performed and landed cleanly, long flight times (i.e., great flight heights) are necessary [4]. Great flight heights depend on an effective jump-off that converts translational energy from the run-up (and preparatory elements) into an optimal amount of translational and rotational energy using the spring properties of the (elastic) surface, which is fundamentally different for each apparatus [5,6].

A long flight time (combined with a correct technique) allows the athletes to perform more rotations around the longitudinal and latitudinal axes and/or to better prepare for the landing. The former increases the difficulty score of the routine, and the latter maximizes the execution score. Neither the difficulty nor the execution of the performed elements during competitive routines may be neglected since the two scores equally influence the final result [7]. In order to succeed in competitions, the difficulty, execution and stability of the performed routines must be at the highest level.

The requirements to perform successfully on each apparatus vary considerably. On the vault, it has been shown by many authors [5,8,9,10,11,12,13] that a high run-up speed within the limited run-up distance (25 m) is crucial to be able to perform difficult vaults. The importance of a high run-up speed is greater for women than for men and depends on the vaulting style (handspring, Tsukahara, Yurchenko). Apparently, run-up speed also influences flight height during the second flight phase. At jump-off and push-off from the springboard and table, the horizontal energy has to be transformed into vertical translational energy and rotational energy [4]. Since women are generally weaker at push-off due to less explosive strength in the upper body, a higher proportion of the total energy must be generated during the run-up and jump-off. In this respect, the importance of the basic conditional abilities is higher for female than male gymnasts [9]. A high run-up speed largely depends on sprinting ability and consequently on the level of peak power and reactive strength of the lower extremities [14,15]. Furthermore, it was shown that an effective jump-off from the springboard has similar characteristics to a drop jump [16] and is closely related to the reactive strength abilities in laboratory conditions [17]. Consequently, a high level of explosive and reactive strength could help gymnasts achieve the necessary run-up speed to execute a powerful jump-off from the springboard, generate sufficient flight height during the second flight phase and therefore have enough flight time to perform difficult vaults and prepare a clean landing.

A competitive floor routine in female artistic gymnastics must be a highly expressive artistic performance that consists of acrobatic elements, gymnastics leaps, turns and jumps, harmoniously choreographed to music [18]. The maximum duration of the routine is 90 s. Kaufmann, et al. [19] have shown that over 60% of the energy during a routine is provided aerobically. Nevertheless, difficult saltos with several rotations around longitudinal and transverse axes and gymnastic jumps with a large jump height must be shown in order to be internationally successful. Effective jump-offs from the elastic floor surface seem to depend strongly on optimal stiffness of the lower extremities. Muscle stiffness serves on the one hand to absorb the impact and on the other hand to transfer the elastic energy of the surface into the body and to convert it into the height of flight, allowing an optimal amount of rotation [20,21]. However, jump-offs to forward saltos are characterized by a higher muscle activation and shorter floor contact times than jump-offs for backward saltos [20]. Therefore, the physical condition for each of these jump-offs should be trained specifically. Generally, there is consensus that muscular power and the optimal utilization of stretch-shortening cycles are key to jump-off effectively from the elastic surface on the floor, to reach sufficient flight height, to perform even more demanding technical skills with perfection [1,22,23] and to prepare a proper landing.

Contrary to the surfaces of the floor and on the vault (springboard), the apparatus “beam” has a nearly non-elastic surface. A competitive routine has a prescribed maximum duration of 90 s and consists of gymnastic jumps, leaps and turns as well as acrobatic elements that have to be performed on the only 10 cm wide apparatus [18]. To perform the acrobatic elements, perfect dynamic balance skills have to be combined with an optimal jump-off. Contrary to the jump-off on the floor or vault, the load distribution on the feet is not symmetrical [24] and must be trained with apparatus-specific drills [25]. Nevertheless, sufficient flight height has to be attained, in order to execute a difficult element and to prepare a clean landing. Most likely, lower body power may play an important role in order to perform these elements also on the beam, but this has not yet been investigated.

In summary, the importance of the physical prerequisites for generating flight height on the different apparatuses for elite athletes, and in particular their development during childhood and adolescence in female gymnasts, is still largely unknown.

Gymnastics is a sport that specializes at a very early age. Beginning training at the age of 5 or 6 has long been the norm, and this is necessary in order to have enough time to learn the large number of different elements. Training continues throughout childhood, adolescence and puberty. The different phases of development have different influences on performance development. During childhood and adolescence, there is an optimal strength–load ratio (i.e., optimal conditions for learning new skills), but this often deteriorates during puberty [26]. As a result, development is not linear and the focus of training needs to change at each stage [27].

To understand the physical and technical developments during childhood and adolescence, athletes’ physical and technical abilities should be monitored regularly during training. New knowledge about the development of individual physical abilities as well as technical development in different age groups will help to provide much more differentiated training recommendations in the future. Consequently, this will help to optimize the training, to avoid overtraining and inefficient training methods and thus to prevent injuries during adolescence.

Therefore, the first aim of our study was to describe the differences between age groups in the physical prerequisites of the lower extremities, and flight height and run-up speed performing basic skills on the floor, beam and vault by young gymnasts between 7 and 15 years of age. The second aim was to calculate the relationship between the physical prerequisites and the flight heights of basic elements on the beam and floor, as well as the run-up speed on the vault.

## 2. Materials and Methods

A total of 33 female athletes between the ages of 7 and 15 (age: 10.7 ± 2.1 y; height: 140 ± 13 cm; body mass: 35.3 ± 9.9 kg) participated in the study. Informed consent was obtained from all participants or their legal guardians, and the study was conducted in accordance with the Declaration of Helsinki and approved by the local Ethics Committee of Canton Bern (Project ID: 2018-00742; 7 June 2018).

At the time of the measurements, all athletes were training in a regional performance center and belonged to national or regional squads. The training times varied according to age between 15 and 28 h per week (7–9 years: 15–20 h; 10–12 years: 20–25 h; 13–15 years: 25–28 h).

The tests were conducted in four different regional performance centers, each on one afternoon. After measurements of the body weight and height, all athletes completed an explosive and a reactive strength test on the force plate, two maximal 20 m sprints, two basic vaults (handspring with landing on a mat stack), three saltos forward on the floor, three saltos forward on the beam, three beam dismounts onto a mat block and three saltos backward including the preparatory elements round-off—back handspring. For this purpose, the athletes were randomly assigned to one of three groups, with one group completing the force plate tests first, the second group completing the saltos on the floor and beam first and the third group completing the 20 m sprints and vaults first. The athletes were instructed to perform at their maximum level in all tests (maximum flight height for the elements on the floor and beam and maximum speed during the 20 m sprint and vault run-up). To avoid fatigue during the tests, the break between trials was set at two minutes and the number of trials per element was set at three.

**Figure 1:**
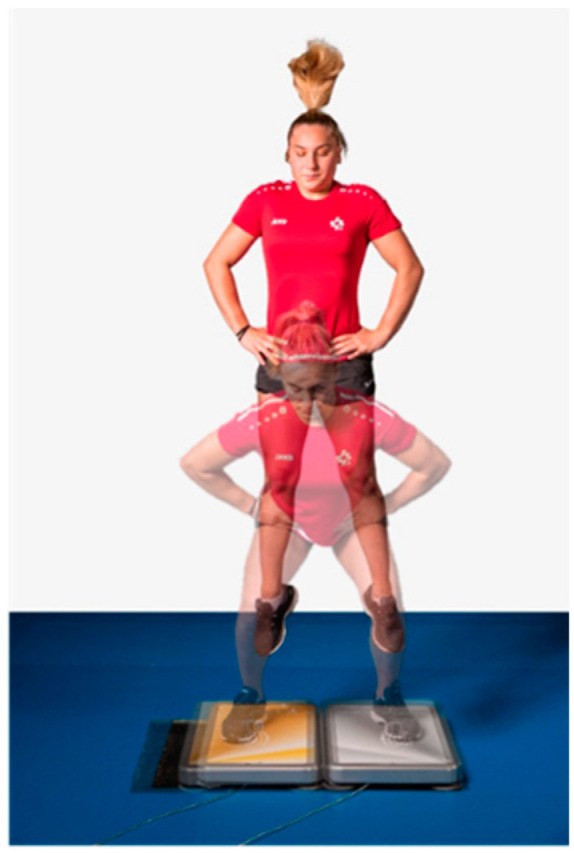
Athlete performing a squat jump on a force plate as part of the explosive strength assessment.

**Figure 2:**
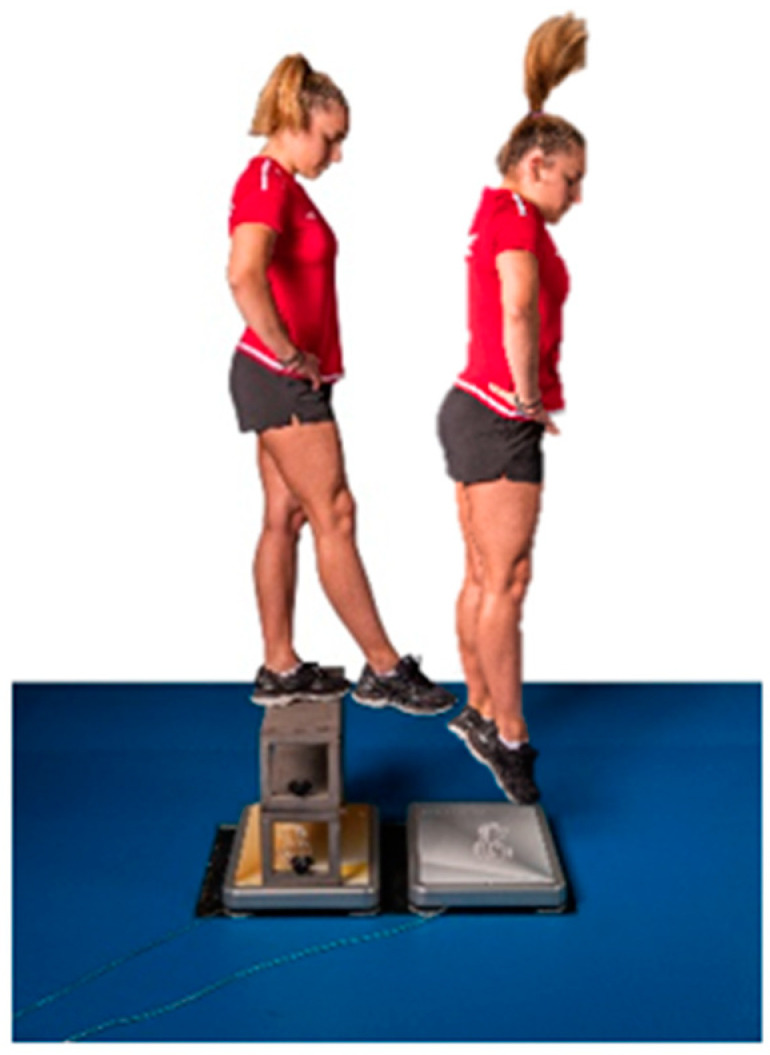
Athlete performing a drop jump from 40 cm onto a force plate as part of the reactive strength assessment.

Explosive (Figure 1) and reactive strength (Figure 2) were determined with a force plate (MLD Test Evo 2, SPSport, Innsbruck, Austria) and the jumps were performed in a standardized manner according to the Swiss Olympic Performance Diagnostics Manual [28]. In the explosive strength test, three countermovement jumps, three squat jumps and three single-leg countermovement jumps with each leg were performed. The relative peak power (Pmax_rel) of each jump was recorded. Coefficients of variation of less than 6.5% are to be expected for standardized measurement procedures such as those described here [29]. In their study, Maier, Gross, Trösch, Steiner, Müller, Bourban, Schärer, Hübner, Wehrlin and Tschopp [28] found measurement errors of less than 4% for double-leg jumps and less than 6% for single-leg jumps. For each jump, the best of the three trials was used for the calculation. For the single-legged CMJ, the average of the left and right sides was used.

In the reactive strength test, two drop jumps from 20, 40 and 60 cm were executed according to the Swiss Olympic Performance Diagnostics Manual [28]. Measurement errors of 2–5% can be expected in standardized drop jump tests [30]. The reactive strength index (jumping height/floor contact time: cm/s) was calculated automatically by the software (Cycess 2.3.4, SpSport, Innsbruck, Austria). Only the maximum value across all drop heights and trials was included in the calculations.

The sprint and vault run-up speed measurements were attained using a laser (LDM301a, Jenoptik, Rostock, Germany). The laser was placed 2 m behind and in line with the vault run-up track. For the maximum 20 m sprints, the vault table was set off to the side. Between the two trials, athletes had time to recover completely (>2 min). The laser gun is a valid and reliable measuring instrument (ICC: 0.99) [31]. It is particularly useful for assessing the maximum speed during a sprint [32].

**Figure 3:**
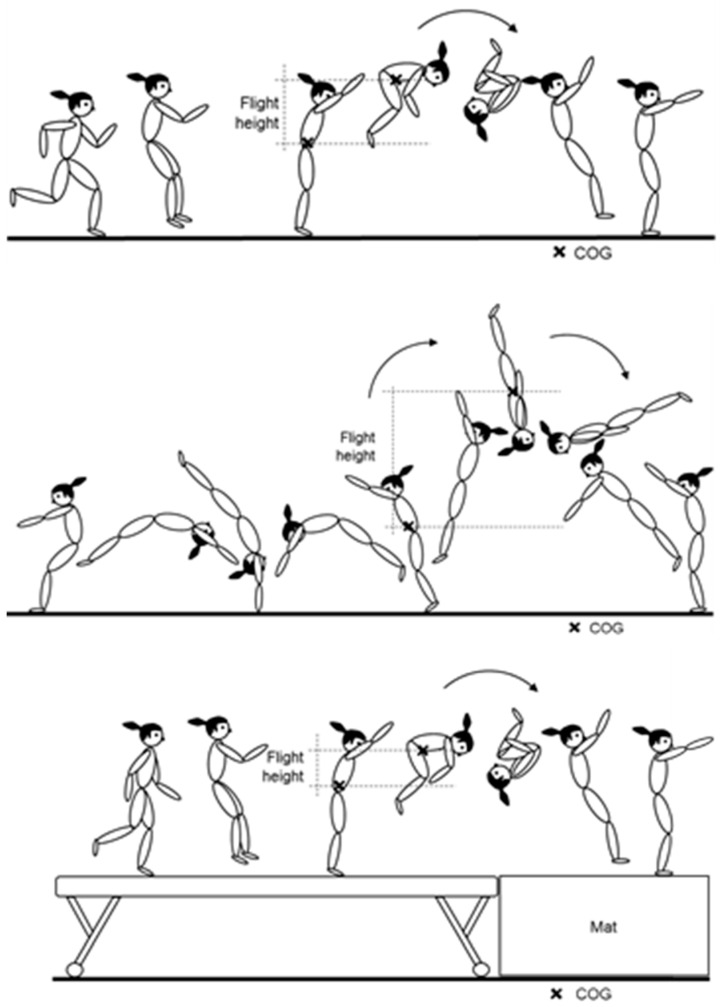
Schematic representation of the determination of the maximum flight height during the forward and backward salto on floor and the forward salto on beam (COG: center of gravity).

The flight heights for saltos on the floor and beam were recorded with an iPad (iPad Pro 9.7″, Apple, Cupertino, CA, USA). The iPad was placed perpendicular to the tumbling track or beam as the height of the body’s center of gravity while standing, and at a distance such that the jump-off and landing were visible on the screen. The maximum flight height (maximum vertical displacement of the body’s center of gravity) was determined using the video analysis software Dartfish (Dartfish SA, Fribourg, Switzerland; Figure 3). Before the measurements, the video setting was calibrated two-dimensionally using a calibration rod [33]. The body’s center of gravity was determined visually at jump-off and at the highest point during flight using the software’s drawing tool. Then, a vertical line between these two points was drawn by using the measurement tool in the software, and the maximum vertical displacement of the body’s center of gravity was calculated automatically according to the previous calibration. This video-based method to assess flight height was considered valid (±3.6% of maximum flight height) and reliable (intrarater reliability: CV% = 0.44%; interrater reliability: CV% = 0.51%) [33].

### Statistical Analysis

The gymnasts in our study were aged between 7 and 15 years and thus formed a very heterogeneous group. In order to avoid the fact that the heterogeneity of age strongly increases the correlation coefficients, age groups were formed, and the analyses were conducted per age group (7–9 y; 10–12 y; 13–15 y). This allowed a more differentiated view of the individual phases of development and to gain age-specific insights that are important for efficient and targeted training. Normal distribution was verified (Shapiro–Wilk test), descriptive statistics were calculated, a Kruskal–Wallis test was used to calculate the differences across all age groups and Mann–Whitney U tests (post hoc) were used to assess differences between the consecutive age groups. Finally, correlations between all parameters were calculated (Spearman’s Rho) separately for each age group. To determine the influence of age on performance on the different tests, the correlation (Spearman’s Rho) between age and all measured parameters was also calculated for all athletes together. The level of significance was set at *p* < 0.05. All calculations were performed using Jasp 0.14.1 Software (Jasp, University of Amsterdam, Amsterdam, The Netherlands).

## 3. Results

In the 7–9 y age group, one athlete could not perform the tests on the force plate due to an injury (unrelated to the measurements). In addition, five athletes could not perform the backward salto on the floor, due to the technical difficulty of this element. Furthermore, in this age group as well as in the age group 13–15 y, two athletes each did not complete the sprint test due to the limited space in one of the gymnasiums.

The mean values (±standard deviation) of all measured parameters are displayed in Table 1.

**Figure 4:**
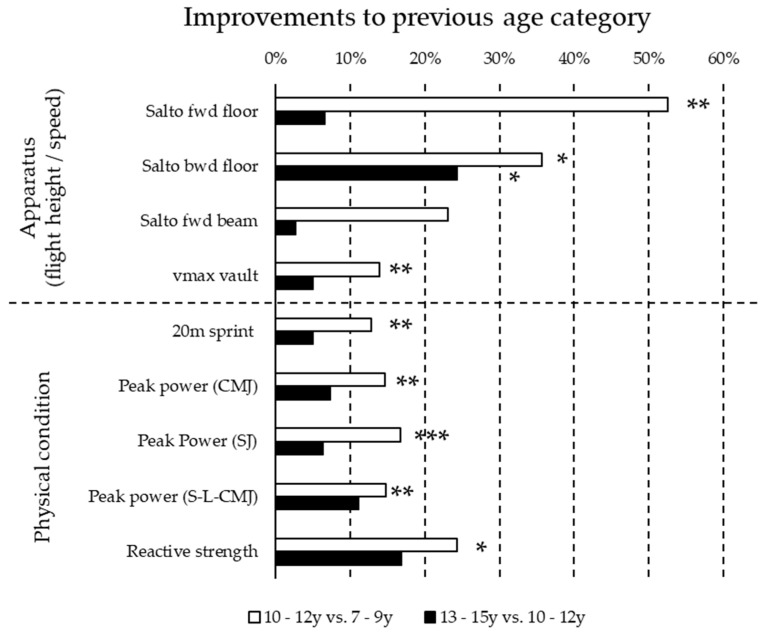
Percentage improvement compared to the previous age group in all technical elements on floor and beam (salto fwd: salto forward; salto bwd: salto backwards) as well as the run-up speed on vault (vmax vault) and all physical condition variables such as the maximum 20 m sprint speed, the peak power on the force plate performing countermovement (CMJ), single-leg countermovement (S-L-CMJ) and squat jumps (SJ) as well as the reactive strength index (significant improvements: * *p* < 0.05; ** *p* < 0.01; *** *p* < 0.001).

The Kruskal–Wallis test showed that with increasing age athletes improved significantly (*p* < 0.01) in all of the measured parameters, except for salto forward on the beam (*p* = 0.20). The differences between the subsequent age groups in percent of all parameters are displayed in Figure 4. Significant differences were observed mostly between the age groups 7–9 y and 10–12 y. The only significant difference between the age groups 10–12 y and 13–15 y was observed for backwards salto on the floor.

**Figure 5:**
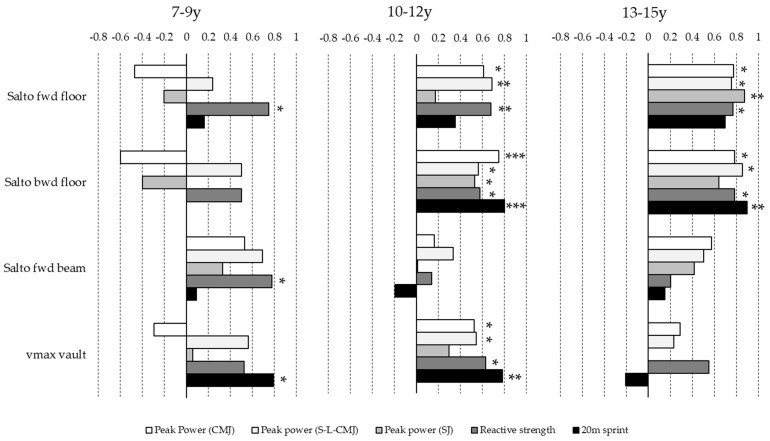
Relationships (Spearman’s Rho) between maximum flight height of the performed elements on floor and beam as well as run-up speed on vault and physical condition variables: peak power of countermovement jumps (CMJ), single-leg countermovement jumps (S-L-CMJ) and squat jumps (SJ), reactive strength and maximum 20 m sprint speed separated in the three age groups (7–9 years: *n* = 9; 10–12 years; *n* = 16; 13–15 years: *n* = 8) (significant correlations: * *p* < 0.05; ** *p* < 0.01; *** *p* < 0.001).

The age of the athletes generally had a highly significant (*p* < 0.001) relationship with performance in all measured parameters (from r = 0.58 (peak power CMJ) to r = 0.77 (20 m sprint)), except for the flight height of salto forward on the beam for which the correlation was low and non-significant (r = 0.26; *p* = 0.14). However, considering the correlations between physical and technical ability separately for each age group, it can be observed that most of the significant relationships were observed for the elements on the floor (age groups 10–12 y and 13–15 y) and vault (age group 10–12 y; Figure 5).

## 4. Discussion

This study investigated differences in the physical condition of lower extremities and the flight height of basic elements on the floor and beam or vault run-up speed in three age groups of female gymnasts between seven and fifteen years of age. Furthermore, the relationships between these parameters were calculated for each age group.

### 4.1. Age-Related Differences in Flight Height, Run-Up Speed and Physical Condition

In general, a continuous increase in all measured parameters from one age group to the next was observed. However, the largest differences were found mostly between age groups 7–9 y and 10–12 y. The considerably smaller differences between the age groups 10–12 y and 13–15 y may be influenced by growth and pubertal development. This may change the power to weight ratio, and it certainly influences the differences in physical condition between the older age groups.

The largest difference between age groups 7–9 y and 10–12 y was found for the maximum flight height of salto forward on the floor (>50%). This skill is one of the first “flight elements” that gymnasts learn during their development due to the low risks and low technical demands when performing it. Therefore, this element can be practiced intensively and perfected at an early stage. This may explain the big difference in the maximum flight height between these two age groups.

The flight height of backwards salto was significantly different across all age groups. The salto backward is a more complex skill which entails some risks when performing it (jump-off backwards, and half of the salto is performed without visual control to the floor). Additionally, in our study, the backwards salto had to be performed from the preparatory elements the round-off and back handspring. With a technically clean execution of these elements, additional energy can be generated and converted into a greater flight height at jump-off. Therefore, not only a single skill but a combination of technically demanding elements had to be performed in order to reach a great flight height. To perform these acrobatic skills effectively and make them technically clean, more training time may be necessary than for the salto forward, which is performed from a short and simple run-up. This may explain the continuous and significant differences in flight height across all age groups. Furthermore, backward acrobatic skills may be of higher importance in the long-term development of future top performances on the floor. The majority of the most difficult elements in the competition routines of top gymnasts are performed with the preparatory elements round-off and back handspring. Moreover, a great flight height in backward salto is necessary to perform a double salto backward. Double back flips are performed more frequently than double front flips during competitive routines. For these reasons, it can be assumed that backward acrobatic elements are trained extensively over a longer period of time, and the flight height must be increased continuously.

The smallest differences of flight height between all age groups were observed for the salto forward on the beam. Our results show an increase in flight height of more than 20% between the age groups 7–9 y and 10–12 y, but almost similar flight heights for the age group 13–15 y. The apparatus beam is only 10 cm wide and, unlike the floor, has no elastic surface. Further, jump-offs on the beam are characterized by a slightly offset foot position. Therefore, the requirements for dynamic balance, precision and technique may prevent maximum jump-off. Our results suggest that due to the high technical requirements, the flight height is only increased to a certain optimum, namely until the athlete reaches a sufficient flight height to land on her feet again. Subsequently, in particular, the technique of the skill is improved while maintaining optimal peak power at jump-off. This “unconscious” strategy may stabilize the technique of execution but not necessarily the flight height of the element. Contrary to this, maximum jump-offs on the beam may have a detrimental effect for the dynamic balance, precision of execution and/or the technique of the element. This could lead to poorer execution or even a fall off the beam or at the landing.

The percentage differences between age groups of the run-up speed on the vault were considerably smaller than differences of the flight height on the floor and beam. Compared to other authors [22], the mean run-up speed of the youngest gymnasts in our study was somewhat smaller (−0.3 m/s) but clearly higher for the age group 13–15 y (+0.7 m/s). Considering the difference between run-up speed on the vault and maximum sprint speed, we found similar differences for all age groups. This is contrary to previous findings in male junior artistic gymnasts, where younger gymnasts had to exploit their speed potential to a higher extent than elite gymnasts when performing their competition vault [14]. In our study, all athletes only had to show a simple handspring. It could be supposed that differences between the run-up speed and maximum sprint speed would be smaller if a competition vault had to have been performed during the measurements. Nevertheless, it can be said that the sprint speed and run-up speed on the vault develop similarly from each age group to the next. Apparently, the run-up speed on the vault increases in a parallel manner to the maximum sprint speed during development.

Considering the development of the physical condition, the largest percentage differences comparing the three age groups were observed in reactive strength. Reactive strength is dependent on neuro-muscular factors such as pre-activation, muscle stiffness, reflex activity and the ability of the tendo-muscular system to store and release energy during the stretch-shortening cycle [34]. Improvements of the stretch-shortening-cycle actions with training can be generally observed during childhood and adolescence [35]. Further, jump-offs in artistic gymnastics training are often characterized by a time pattern that is similar to drop jumps [22,36]. Consequently, reactive jump-offs are performed (and trained) in technical training on a daily basis. This may explain the continuous development of reactive strength.

Interestingly, we found clearly smaller differences between age groups for explosive strength (peak concentric jumping power) than for reactive strength. Peak power is considered an important physical condition in women’s artistic gymnastics [3,37,38]. In contrast to the frequently occurring reactive jumps during technical training, only a few elements in technical training are performed with a jump-off resembling the slow stretch-shortening cycle typical of both CMJ and SJ. Hence, it could be supposed that explosive strength is not sufficiently trained during development. However, peak power (and its prerequisites: quickness, speed strength and maximum strength) should be systematically integrated in addition to technical training. In particular, maximal initial horizontal accelerations on the floor and on the vault may strongly depend on the level of peak power of the lower extremities. Consequently, it may help athletes to generate more horizontal acceleration in a shorter time, and therefore greater horizontal kinetic energy, which can be converted into flight height at the jump-off on the floor and vault.

Apart from reactive strength, the most continuous development across the age groups was observed in the peak power of the single-legged CMJ. In artistic gymnastics, many skills (in particular, gymnastic jumps on the floor and beam) are executed with a single-leg jump-off. Therefore, unilateral jump-offs (similar to reactive jump-offs) are performed daily in technical training. In order to be able to generate enough flight height for difficult jumps, the ability to effectively execute a single-leg jump-off must be developed continuously. In addition, the run-ups for the acrobatic elements on the floor, as well as on the vault, are characterized by unilateral muscle actions. This may explain the continuous development of this parameter. Thus, a high level of single-leg peak power is a fundamental physical condition in women’s artistic gymnastics.

### 4.2. Correlations between Physical Condition, Flight Heights and Run-Up Speed

As might be expected, age was significantly correlated with performance on all parameters in all tests (except the beam). In order to be able to discuss the influence of age in a more differentiated way, the correlations in the different age groups are particularly taken into account in this discussion.

Across the three age groups, the strongest correlations between the physical condition and flight heights on the apparatus were found for the age group 13–15 y. Contrary to this, most of the significant correlations were found in the age group 10–12 y, most probably due to the higher number of athletes in this age group. Generally, the lowest and most divergent correlations were observed in the age group 7–9 y. In order to be able to perform skills in gymnastics, the utilization of the physical condition must be optimally adapted to the individual technical level. Moreover, the methodologically correct training of the technique may lead to a reduction in anxiety and improve self-confidence in one’s own abilities. The better the technique and the less anxious the athlete, the more efficiently the physical condition can be utilized to perform an element. The less substantial an athlete’s training experience, the less accurately the physical condition may contribute to the realization of the technique, which may explain our general findings.

Considering the correlations between the physical condition variables and the flight height of a salto forward on the floor, our results show that for the age group 7–9 y, only reactive strength seemed to be important to achieve a great height of flight. Contrary to this, we found only positive correlations (mostly r > 0.6) between all physical prerequisites and flight height for the older age groups. For gymnasts in the age group 7–9 y, performing a salto forward on the floor was certainly a challenge. In order to achieve more control during the execution of an element, gymnasts generally have to diminish the speed of execution. Consequently, the run-up for a salto forward may be executed rather slowly and adapted to the individual technical level. Consequently, a great proportion of the flight height must be generated with the reactive take-off from the elastic surface. This may explain why (only) reactive strength was strongly correlated with the flight height of a salto forward on the floor in this age group. Nevertheless, the level of reactive strength seemed to be crucial to generate the flight height for a salto forward on the floor. These results are in line with other studies that found very short contact times [36] at jump-offs for forward elements on the floor and a strong relationship between the reactive strength level and competition results [39]. In contrast to the youngest gymnasts in our study, the better technical and physical abilities of older gymnasts permitted them to use their entire physical potential, which may explain the strong correlations with all measured physical conditions.

For the salto backward on the floor, our results show that strategies to generate a maximum flight height may change across the age groups. For the age group 7–9 y, we found no strong correlation between the flight height and physical condition (probably due to the unstable technique of the preparatory elements round-off and back handspring and inefficient jump-off). For the age group 10–12 y, a high horizontal acceleration seemed to be crucial, but for the age group 13–15 y, the importance of the level of peak power may increase in order to generate the height of flight. Our results may be explained with the finding of Freyler, Ritzmann, Fuhrmann and Gollhofer [39], who stated that by increasing training experience, the precision of neuronal control of the lower extremity muscles during the floor jump-off improves, leading to a better utilization of the elastic properties of the floor and resulting in greater flight heights. Therefore, muscular and neural control must be perfectly matched to the spring characteristics of the elastic surface. Until this is the case, physical characteristics other than jumping abilities are seemingly more important to generate the maximum flight height. In addition, performing the acrobatic combination of a run-up, round-off, back handspring and salto backwards on the floor requires the gymnasts to convert a part of the horizontal translation energy of the run-up into rotational energy for the execution of the preparatory elements round-off and back handspring, and reconvert the energy into an optimal amount of flight height and rotation at jump-off for the salto backwards. More skilled athletes may be able to generate additional kinetic energy during the preparatory elements which may result in higher forces at the jump-off from the elastic surface [21]. The higher initial vertical force and higher body mass of older athletes increase the spring amplitude of the elastic surface, which entails a longer ground contact time [36], seemingly generating a jump-off that is most closely related to CMJ. Nevertheless, to use the elasticity of the floor, a high muscle stiffness in lower extremities is required [36,39]. This may explain the medium to high correlations across all age groups between the flight height and reactive strength.

Compared to the other apparatuses, we found generally lower correlations between the flight height of the salto forward and physical conditions on the beam. However, this was not the case for the 7–9 y age group, for which at least all correlations between the physical condition and flight height were positive. Generally, the strongest correlations were found between the flight height and peak power of the S-L-CMJ across all age groups. The beam is only 10 cm wide. Jump-offs from the beam are executed with one foot behind the other, and the run-up speed is reduced due to the limited run-up distance and the high requirements on dynamic balance during the run-up. These factors may reduce the effectivity of the jump-off [40]. Nevertheless, the slightly offset foot position at jump-off seems to provoke a jump-off that is mainly dependent on the level of single-leg peak power. This finding is in line with Pajek, Hedbávný, Kalichová and Čuk [24], who found the unilateral distribution of the load during jump-offs from the beam. This may show that an effective jump-off from the beam has very special requirements, which should be trained separately in physical training. Furthermore, it is also interesting to note that peak power of the lower extremities during long stretch-shortening cycles tends to correlate more strongly with the flight height of a salto forward on the beam than does reactive strength. Due to the hard and almost inelastic surface of the beam, it could be suspected that rather short contact times are beneficial for effective jump-offs. However, to maintain control over the dynamic balance during jump-off on the narrow jumping surface, athletes reduce the execution velocity of the movement, which may result in a longer contact time. This could explain the strong correlations with peak power values of the lower extremities. These results are in line with the findings of Potop, et al. [41], who pointed out the importance of a proper technique in order to perform difficult dismounts on the beam.

On the vault, our results show that across all age categories, the level of reactive strength seemed to be crucial for a high run-up speed. Our results are in line with the findings of other authors who have found that a high level of explosive and reactive strength is important for a high run-up speed on the vault [14]. Namely, a high level of explosive strength enables athletes to accelerate [42,43], and a high reactive strength level allows them to achieve high maximum speed [44,45]. Since run-up on the vault is a standardized sprint within the limited run-up distance (25 m) [22], athletes mainly reach a high run-up speed by increasing step frequency rather than step length during the run-up [14]. The high step frequencies can only be achieved by a short ground contact time, which is closely related to the level of reactive strength [46]. Furthermore, the run-up speed was strongly correlated to the maximum sprint speed for the age categories 7–9 y and 10–12 y. Other authors have shown similar correlations [14] in men’s artistic gymnastics. In order to achieve a high run-up speed on the vault, athletes must be able to generate high speed during linear sprints. Therefore, sprinting speed and sprinting technique should be trained separately during training. Contrary to the findings in the younger age groups, the run-up speed was not positively correlated to the sprint speed in the age group 13–15 y. This is probably related to the fact that the task (simple handspring) was not sufficiently difficult for these athletes, and they may have reduced their run-up speed accordingly.

## 5. Conclusions

In women’s artistic gymnastics, jumping ability is of crucial importance, since three of the four apparatuses require a high level of lower body power. In order to perform difficult elements, land cleanly and thereby obtain a high final score in a competition, a long flight time (i.e., a large flight height) is necessary.

The results of the study show that across the different age groups, a parallel development of the physical condition and the sport-specific technique (movement learning) took place.

In addition, the correlations between the physical condition (peak power, reactive strength, sprint speed) and flight height, as well as the run-up speed on the apparatus, were shown. The strongest correlations were found on the floor, since jumping abilities can be applied almost directly, but lower on the beam due to the coordinatively very demanding jump-off on the narrow beam and on the vault due to the necessary transfer of vertical jump-off abilities into (horizontal) sprint speed.

If the tests presented in this study are used regularly as screening across age groups, training recommendations can be derived not only for lower body peak power and reactive strength and/or technique training, but also for assessing the relationships between physical conditioning and technical training.

## Figures and Tables

**Table 1:** Mean values (±standard deviation) of the flight height (maximum displacement of body’s center of gravity) for the performed elements on floor and beam (salto forward (fwd) and backwards (bwd)) as well as peak power at countermovement (CMJ), single-leg countermovement (S-L-CMJ) and squat jump (SJ) and reactive strength (reactive index (RI 1: jumping height/floor contact time)) of the lower extremities.

		7–9 Years	10–12 Years	13–15 Years
	*n*	9	16	8
	Age (y)	8.11 ± 0.78	10.75 ± 0.78	13.38 ± 0.74
	Height (m)	1.28 ± 0.09	1.41 ± 0.09	1.57 ± 0.05
	Body mass (kg)	26.22 ± 4.42	34.57 ± 6.62	47.34 ± 7.72
Flight height floor	Salto fwd (m)	0.42 ± 0.13	0.65 ± 0.12	0.69 ± 0.11
	Salto bwd (m)	0.52 ± 0.14	0.71 ± 0.11	0.88 ± 0.15
Flight height beam	Salto fwd (m)	0.19 ± 0.03	0.23 ± 0.09	0.23 ± 0.09
Run-up speed vault	vmax (m/s)	5.75 ± 0.54	6.54 ± 0.40	6.87 ± 0.32
20 m sprint speed	vmax (m/s)	6.06 ± 0.26	6.83 ± 0.30	7.17 ± 0.33
Explosive strength	CMJ (W/kg)	40.06 ± 3.72	45.94 ± 4.31	49.30 ± 7.34
	S-L-CMJ (W/kg)	23.87 ± 2.79	27.40 ± 2.79	30.44 ± 3.69
	SJ (W/kg)	37.19 ± 3.14	43.40 ± 4.01	46.13 ± 5.91
Reactive strength	RI 1 (cm/s)	15.14 ± 1.71	18.82 ± 4.00	22.00 ± 4.18

## Data Availability

Not applicable.
